# Efficacy and safety profile of COVID-19 vaccine in cancer patients: a prospective, multicenter cohort study

**DOI:** 10.2217/fon-2021-1248

**Published:** 2022-01-27

**Authors:** Ayse Irem Yasin, Sabin Göktas Aydin, Bilge Sümbül, Lokman Koral, Melih Şimşek, Çağlayan Geredeli, Akın Öztürk, Perihan Perkin, Derya Demirtaş, Engin Erdemoglu, İlhan Hacıbekiroglu, Emre Çakır, Eda Tanrıkulu, Ezgi Çoban, Melike Ozcelik, Sinemis Çelik, Fatih Teker, Asude Aksoy, Sedat T Fırat, Ömer Tekin, Ziya Kalkan, Orhan Türken, Bala B Oven, Faysal Dane, Ahmet Bilici, Abdurrahman Isıkdogan, Mesut Seker, Hacı M Türk, Mahmut Gümüş

**Affiliations:** ^1^Bezmialem Vakif University, Department of Medical Oncology, Istanbul 34093, Turkey; ^2^Medipol University, Department of Medical Oncology, Istanbul 34214, Turkey; ^3^Bezmialem Vakif University, Department of Microbiology, Istanbul 34093, Turkey; ^4^Canakkale 18 March University, Department of Medical Oncology, Canakkale 17020 ,Turkey; ^5^Okmeydani Training and Research Hospital, Department of Medical Oncology, Istanbul 34384, Turkey; ^6^SureyyapasaChest Dıseases And Thoracıc Surgery Traınıng And Research Hospıtal, Department of Medical Oncology, Istanbul 34844, Turkey; ^7^Yildirim Beyazit University Yenimahalle Training and Research Hospital, Department of Medical Oncology, Ankara 06330, Turkey; ^8^AnkaraCity Hospital, Department of Medical Oncology, Ankara06800, Turkey; ^9^GöztepeMedeniyet University, Department of Medical Oncology, Istanbul 34000, Turkey; ^10^Sakarya University Medicine Faculty, Departmentof Medical Oncology, Sakarya 54050, Turkey; ^11^Haydarpasa Training and Research Hospital, University of Health Sciences, Istanbul 34668, Turkey; ^12^Marmara University School of Medicine, Department of Medical Oncology, Istanbul 34722, Turkey; ^13^Istanbul Oncology Hospital, Department of Medical Oncology, Istanbul 34846, Turkey; ^14^Gaziantep University, Department of Medical Oncology, Gaziantep 27470, Turkey; ^15^Fırat University Faculty of Medicine, Department of Medical Oncology, Elazıg 23119, Turkey; ^16^Erciyes University, Department of Medical Oncology, Kayseri 38039, Turkey; ^17^InönüUniversity, Department of Medical Oncology, Malatya 44280, Turkey; ^18^DicleUniversity, Department of Medical Oncology, Diyarbakır 21200, Turkey; ^19^MaltepeUniversity, Department of Medical Oncology, Istanbul 34844, Turkey; ^20^Bahcesehir University School of Medicine, Department of Medical Oncology, Istanbul 34349, Turkey; ^21^Acıbadem University, Department of MedicalOncology, Istanbul 34758, Turkey

**Keywords:** cancer, chemotherapy, CoronaVac, COVID-19, COVID-19 vaccines, immunotherapy, malignancy, SARS-CoV-2

## Abstract

**Aim:** To compare the seropositivity rate of cancer patients with non-cancer controls after inactive SARS-CoV-2 vaccination (CoronaVac) and evaluate the factors affecting seropositivity. **Method:** Spike IgG antibodies against SARS-CoV-2 were measured in blood samples of 776 cancer patients and 715 non-cancer volunteers. An IgG level ≥50 AU/ml is accepted as seropositive. **Results:** The seropositivity rate was 85.2% in the patient group and 97.5% in the control group. The seropositivity rate and antibody levels were significantly lower in the patient group (p < 0.001). Age and chemotherapy were associated with lower seropositivity in cancer patients (p < 0.001). **Conclusion:** This study highlighted the efficacy and safety of the inactivated vaccine in cancer patients.

Clinical Trials Registration: NCT04771559 (ClinicalTrials.gov)

## Plain language summary

Cancer patients are at high risk for infection with SARS-CoV-2 and of developing the associated disease, COVID-19, which therefore puts them in the priority group for vaccination. This study evaluated the efficacy and safety of CoronaVac, an inactivated virus vaccine, in cancer patients. The immune response rate, defined as seropositivity, was 85.2% in the cancer patient group and 97.5% in the control group. The levels of antibodies, which are blood markers of immune response to the vaccine, were also significantly lower in the patient group, especially in those older than 60 years and receiving chemotherapy. These results highlight the importance of determining the effective vaccine type and dose in cancer patients to protect them from COVID-19 without disrupting their cancer treatment.

COVID-19, which emerged in China in 2019 and spread all over the world in a short time, caused many deaths around the world [1]. In many countries, including Turkey, measures are continuing to prevent the spread of the virus, which has many negative effects on social and economic life. Since the beginning of the pandemic, many countries have carried out studies to develop a vaccine against COVID-19. Today there are more than ten different vaccines currently in use worldwide [2]. Turkey’s national immunization program continues by prioritizing high-risk groups such as elderly adults and cancer patients. Approximately 70% of the population has been vaccinated with at least two doses [3].

Studies have shown that the morbidity and mortality of COVID-19 in cancer patients are higher than in non-cancer individuals [4–6]. COVID-19 progresses more severely in cancer patients due to the natural course of the cancer and the oncological treatments [7,8].

Cancer patients were also negatively affected by disruptions in cancer diagnosis and treatment during the pandemic. A European survey showed an average reduction of 29.3% in all types of oncological surgeries [9]. Riera *et al.* reviewed delays and disruptions in cancer management due to the pandemic; they reported up to 77.5% interruption in any stage of cancer treatment [10]. As a result of interruptions in oncological diagnosis and treatment processes, the increase in cancer-related deaths in England over the past year was estimated to be 20% [11].

The COVID-19 seroprevalence in cancer patients was evaluated in recent studies. Fillmore *et al.* screened the results of 22,914 cancer patients tested for COVID-19 and reported 7.8% positivity [12]. In another study, 928 cancer patients with a COVID-19 diagnosis were evaluated, and 4% were reported as asymptomatic [13]. The leading oncological societies, such as the American Society of Clinical Oncology, European Society of Medical Oncology and National Comprehensive Cancer Network (NCCN), have developed guidelines to minimize the negative effects of the COVID-19 pandemic on cancer patients. However, there is no consensus for SARS-CoV-2 testing of asymptomatic patients before initiation of immunosuppressive therapies [14]. An individual risk–benefit assessment for each patient appears to be the most reliable method yet [14].

Because there is no standard treatment for COVID-19, vaccination is considered to be the cornerstone for mitigation of the pandemic. The severe course of COVID-19 in cancer patients puts them among the priority groups for vaccination. The NCCN recommends that people with active cancer undergoing treatment, those about to be treated for cancer and those who have been treated for cancer in the past 6 months should be prioritized to receive vaccinations as soon as possible [15]. Different types of COVID-19 vaccines are currently available around the world. CoronaVac, an inactivated vaccine, is one of the most applied vaccines. Solodky *et al.* reported that the antibody level in cancer patients after COVID-19 was lower than that in healthy individuals [16]. A similar situation is expected to be seen in the post-vaccine antibody response. Although the seroconversion rate in healthy adults after two doses of inactivated vaccine was reported as 100% in the CoronaVac study, seroconversion in cancer patients was not assessed [17]. In another study evaluating the efficacy of CoronaVac, the seropositivity rate was 89.7% [18]. Furthermore, the seroconversion rate of the BNT162b2 mRNA vaccine was found to be 95% in healthy adults [19]. Currently, limited data are available showing the efficacy and safety of COVID-19 vaccines in cancer patients. Ariamanesh *et al.* recently demonstrated 86.9% seropositivity after administration of inactivated vaccine in patients with malignancy [20]. Massarweh *et al.* reported 90% seropositivity in 102 cancer patients vaccinated with the BNT162b2 mRNA vaccine [21]. However, the role of COVID-19 vaccination remains a challenging issue in cancer patients.

In this study we aimed to compare cancer patients with non-cancer controls in terms of the efficacy and safety of inactive SARS-CoV-2 (CoronaVac) vaccination. In addition, factors affecting seropositivity in cancer patients were evaluated.

This trial is registered with ClinicalTrials.gov (NCT04771559) and is closed to accrual.

## Patients & methods

### Study design

This study is a prospective, multicenter cohort study evaluating the efficacy and safety of the CoronaVac in cancer patients. Initially, 2154 adult patients with histologically diagnosed solid tumors who were admitted to medical oncology clinics between 1 March and 1 July 2021 were informed about the study; the control group consisted of healthcare workers and volunteers accompanying the patients. From this initial group, 776 cancer patients and 715 non-cancer volunteers who received a second dose of inactivated vaccine in 4–6 weeks were included in the study. Vaccination information and the COVID-19 history of the participants were checked from the national health record database. Patients and controls who had a documented COVID-19 infection (positive PCR test result) at any time before enrollment and patients who received an mRNA vaccine were excluded. In addition, controls who were pregnant or had an immunosuppressive disease or were receiving immunosuppressive therapy for any reason were excluded from the study. The study was carried out with permission of the Turkish Ministry of Health and approved by the local ethics committee (02/28). All participants signed a written informed consent form.

### Assessments

Blood samples were taken from the patients and centrifuged at 2500 rpm for 10 min. The separated serum samples were backed up in two Eppendorf tubes and stored at -80 or -20°C. All serum samples were delivered by cold chain and collected in a single center. A US FDA-approved chemiluminescent microparticle immunoassay, the Abbott Architect i1000sr SARS-CoV-2 IgG II Quant assay (Abbott Laboratories, IL, USA), was used to quantify IgG antibodies against the SARS-CoV-2 spike receptor-binding domain following the manufacturer’s instructions [22]. This assay has 98.1% sensitivity and 99.6% specificity at least 15 days after first symptom onset or documented COVID-19 infection [23]. An IgG level ≥50 AU/ml is accepted as seropositive.

Patient characteristics were collected and included age, sex, BMI, smoking status, comorbidities and receipt of any other vaccination (influenza or pneumococci) within 2 years. All participants were asked about local and systemic side effects of vaccination. Additionally, all clinical information about the cancer diagnosis (tumor type, disease stage and treatment status) were recorded. Treatment groups were: chemotherapy group (including taxane, platin, fluorouracil, gemcitabine, anthracycline, cyclophosphamide, pemetrexed); immunotherapy group (including nivolumab, pembrolizumab and atezolizumab); targeted therapies group (tyrosine kinase inhibitors, anti-VEGF agents, trastuzumab, pertuzumab, CDK4/6 inhibitors); and hormonal therapies group (tamoxifen, aromatase inhibitors, LHRH analogs). We evaluated each treatment group for seropositivity. Additionally, we created another group for those receiving active targeted or immunotherapies and compared the seropositivity rates of this group with those of the active chemotherapy group.

### Statistical analysis

Descriptive statistics are shown as mean ± standard deviation for variables with normal distribution, median (minimum to maximum) for non-normal distributions, and the number of cases and percentage (%) for nominal variables. The Mann–Whitney U-test was used for comparison of the groups. Pearson’s χ-square or Fisher’s exact tests were performed for nominal variables. Multivariate analysis was applied with a logistic regression test. A p-value < 0.05 was considered to be statistically significant. SPSS for Windows (v. 22; IBM Corp., NY, USA) was used to analyze the data.

## Results

Our study group consisted of 776 cancer patients and 715 non-cancer controls. The median age in the patient group was 64 years (range: 20–88), and the median age in the control group was 50 years (range: 21–94). The characteristics of the study participants are shown in Table 1.

**Table 1.** Characteristics of study participants.

Characteristic	Patient group (n = 776)		Control group (n = 715)		p-value
	n	(%)	n	(%)	
Age, median (range)	64 (20–88)		50 (21–94)		<0.001^†^
**Age (years)** <60 ≥60	291 485	37.5 62.5	614 101	85.9 14.1	<0.001^†^
**Sex** Female Male	433 343	55.8 44.2	398 317	55.7 44.3	0.958
BMI, median (range)	27.1 (16–48)		26.1 (18–40)		0.943
**BMI** <25 kg/m^2^ ≥25 kg/m^2^	187 422	30.7 69.3	118 269	30.5 69.5	0.943
**Smoking** No Ex-smoker Yes	436 165 135	59.3 22.4 18.3	428 16 149	72.2 2.7 25.1	<0.001^†^
**Diabetes mellitus** No Yes	635 141	81.8 18.2	666 49	93.1 6.9	<0.001^†^
**Hypertension** No Yes	513 263	66.1 33.9	616 99	86.2 13.8	<0.001^†^
**Coronary disease** No Yes	710 66	91.5 8.5	698 17	97.6 2.4	<0.001^†^
**Chronic renal failure** No Yes	759 17	97.8 2.2	714 1	99.9 0.1	<0.001^†^
**Chronic liver disease** No Yes	761 15	98.1 1.9	709 6	99.2 0.8	0.081
**Rheumatological disease** No Yes	766 10	98.7 1.3	707 8	98.9 1.1	0.816
**Psychiatric disease** No Yes	762 14	98.2 1.8	713 2	99.7 0.3	0.004^†^
**Respiratory disease** No Yes	741 35	95.5 4.5	703 12	98.3 1.7	0.002^†^
**Other** No Yes	731 45	94.2 5.8	686 29	95.9 4.1	0.152

† Statistically significant results.

The seropositivity rate was 85.2% and the median antibody titer was 363.9 AU/ml in the patient group. The seropositivity rate was 97.5% and the median antibody titer was 656.5 AU/ml in the control group. When the two groups were compared, the seropositivity rate and antibody levels were significantly lower in the patient group than in the non-cancer controls (p < 0.001). Additionally, administration of influenza and pneumococcal vaccine prevalence was higher in the patient group (p < 0.001). Vaccine features and antibody levels are shown in Table 2.

**Table 2.** Vaccine features and antibody levels of the study population.

	Patient group (n = 776)		Control group (n = 715)		p-value
	n	(%)	n	(%)	
Antibody level, median (range)	363.9 AU/ml (0–40,000)		656.5 AU/ml (0.2–10,615.3)		<0.001^†^
**Seropositivity** Positive (≥50) Negative (<50)	661 115	85.2 14.8	697 18	97.5 2.5	<0.001^†^
**Other vaccines** Yes No	217 559	28.0 72.0	117 598	16.4 83.6	<0.001^†^
**Type of vaccine** Influenza Pneumococcal Influenza + pneumococcal	71 59 87	32.7 27.2 40.1	48 24 45	41.0 20.5 38.5	0.236

† Statistically significant results.

While the incidence of side effects after the first dose of vaccine was 15.9% in the patient group, this rate was 22.5% in the control group. The rate of side effects reported after the first dose was significantly higher in the controls than the patients (p = 0.001). While the most common side effect in the control group was local pain (9.7%), the most common side effect in the patient group was fatigue (6.4%). When the prevalence of side effects after the second dose was compared, there was no significant difference between the two groups (Table 3).

**Table 3.** Side effects after the first and the second doses of the vaccine.

Characteristics	Patient group (n = 776)				Control group (n = 715)				p-value
	Total (%)	Gr 1 (%)	Gr 2 (%)	Gr 3–4 (%)	Total (%)	Gr 1 (%)	Gr 2 (%)	Gr 3–4 (%)	
First dose	15.9				22.5				0.001^†^
Local pain	5.7	5.3	0.4	–	9.7	8.3	1.1	0.3	0.005^†^
Erythema	0.5	0.4	0.1	–	2.1	1.8	0.3	–	0.009^†^
Fever	2.1	1.2	0.8	0.1	1.8	1.7	–	0.1	0.852
Fatigue	6.4	5.0	1.3	0.1	8.4	6.7	1.4	0.3	0.165
Headache	4.6	3.6	0.9	0.1	7.8	6.2	1.1	0.6	0.013^†^
Myalgia	4.5	3.2	0.9	0.4	6.7	4.4	2.2	0.1	0.071
Nausea	1.8	1.7	–	0.1	1.4	1.4	–	–	0.681
Diarrhea	0.8	0.4	0.4	–	1.0	0.8	0.2	–	0.783
Other	0.9	0.9	–	–	2.6	2.6	–	–	0.269
Second dose	15.2				16.8				0.436
Local pain	5.0	4.6	0.4	–	7.7	6.2	1.3	0.3	0.042^†^
Fever	1.3	1.0	0.3	–	2.1	2.0	0.1	–	0.234
Fatigue	6.7	4.9	1.5	0.3	6.4	5.1	1.0	0.3	0.917
Headache	4.5	3.7	0.8	–	4.8	3.5	0.7	0.6	0.902
Myalgia	4.9	3.4	1.0	0.5	5.9	4.3	1.0	0.6	0.422
Nausea	1.2	0.8	0.4		0.7	0.7	–	–	0.427
Diarrhea	0.9	0.7	0.1	0.1	0.3	0.3	–	–	0.182
Other	1.1	1.1	–	–	1.3	1.3	–	–	0.647

† Statistically significant results.

Gr: Grade.

**Figure 1.**
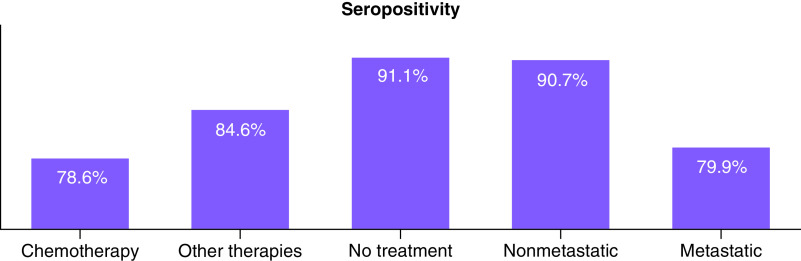
Seropositivity rates of cancer patients after SARS-CoV-2 vaccination according to the treatment status and stage of the disease.

The most common tumor types were breast cancer (32.3%), lung cancer (23.6%), gastrointestinal cancer (22.4%) and genitourinary cancer (13.8 %). Of the patients, 51.3% (n = 398) had metastatic disease; 39.8% (n = 309) were on active chemotherapy; 15.1% (n = 117) were on immunotherapy or targeted therapies; and 45.1% (n = 350) had not received any of these treatment modalities within the previous 3 months. The seropositivity rates were 78.6% in the active chemotherapy group, 85.7% in the immunotherapy group, 86.0% in the targeted therapies group and 87.1% in the hormone therapy group. For the patients not receiving any active treatment including chemotherapy, immunotherapy or targeted therapies, the seropositivity rate was 91.1% (Table 4). Additionally, 90.7% of the nonmetastatic patients and 79.9% of the metastatic patients were seropositive (Figure 1).

**Table 4.** The factors affecting seropositivity in the study population.

Factors affecting seropositivity in the patient group (univariate analysis)			
Characteristics	n (%)	*Seropositivity (%)*	p-value
**Age (years)** <60 ≥60	291 (37.5) 485 (62.5)	93.5 80.2	<0.001^†^
**Gender** Female Male	433 (55.8) 343 (44.2)	88.0 81.6	0.015^†^
**BMI** <25 kg/m^2^ ≥25 kg/m^2^	187 (30.7) 422 (69.3)	88.8 86.3	0.435
**Smoking** No Ex-smoker Yes	436 (59.2) 165 (22.4) 112 (17.8)	85.3 87.3 83.0	0.577
**Tumor type** Breast Gastrointestinal Genitourinary Lung Other	251 (32.3) 174 (22.4) 107 (13.8) 183 (23.6) 61 (7.9)	88.0 86.2 84.1 80.9 85.2	0.335
**Treatment type (active)** No treatment Chemotherapy Targeted or IO	350 (45.1) 309 (39.8) 117 (15.1)	91.1 78.6 84.6	<0.001^†^
**Chemotherapy** Never Not in the last 3 months Active	152 (19.6) 315 (40.6) 309 (39.8)	87.5 90.5 78.6	<0.001^†^
**Immunotherapy (IO)** Yes No	42 (5.4) 734 (94.6)	85.7 85.1	0.920
**Targeted therapies** Yes No	178 (22.9) 598 (77.1)	86.0 84.9	0.811
**Hormone therapy** Yes No	209 (26.9) 567 (73.1)	87.1 84.5	0.426
**Comorbidities** No Yes	373 (48.1) 403 (51.9)	86.9 83.6	0.225
**Stage** Nonmetastatic Metastatic	378 (48.7) 398 (51.3)	90.7 79.9	<0.001^†^

Factors affecting seropositivity in the control group (univariate analysis)			
Characteristics	n (%)	*Seropositivity (%)*	p-value
**Age (years)** <60 ≥60	614 (85.9) 101 (14.1)	98.4 92.1	<0.001^†^
**Gender** Female Male	398 (55.7) 317 (44.3)	98.0 96.8	0.347
**BMI** <25 kg/m^2^ ≥25 kg/m^2^	118 (30.5) 269 (69.5)	97.5 97.0	0.435
**Smoking** No Ex-smoker Yes	428 (72.2) 16 (2.7) 149 (25.1)	97.2 93.8 97.3	0.711
**Comorbidities** No Yes	544 (76.1) 171 (23.9)	97.8 96.5	0.399

† Statistically significant results.

IO: Immunotherapy.

In univariate analysis of the patient group, chemotherapy, metastatic disease, age and male gender were negatively correlated with seropositivity (p < 0.001). The seropositivity rate in the active chemotherapy group was significantly lower than in the group of patients not receiving active chemotherapy (p < 0.001). Tumor type, BMI, smoking and comorbidities were not associated with seropositivity (Table 4). In univariate analysis of the control group, age was found to be the only factor negatively correlated with seropositivity (p < 0.001; Table 4). When the multivariate analysis was performed, age and chemotherapy were defined as the factors significantly associated with lower seropositivity in cancer patients (p < 0.001 and p = 0.038, respectively ; Tables 5 & 6).

**Table 5.** The factors affecting seropositivity in the study population (multivariate analysis).

Characteristics	SE	RR	95% CI	p-value
Noncancer vs cancer	0.286	3.519	2.009–6.162	<0.001^†^
Age (<60 vs ≥60)	0.246	3.545	2.190–5.737	<0.001^†^
Gender (female vs male)	0.194	1.271	0.868–1.859	0.218
Comorbidities (yes vs no)	0.195	1.129	0.771–1.655	0.533

† Statistically significant results.

RR: Relative risk; SE: Standard error.

**Table 6.** The factors affecting seropositivity in the patient group (multivariate analysis).

Characteristics	SE	RR	95% CI	p-value
Age (<60 vs ≥60)	0.276	3.016	1.758–5.176	<0.001^†^
Gender (female vs male)	0.221	1.154	0.701–1.667	0.724
Chemotherapy (yes vs no)	0.358	1.396	0.692–2.818	0.038^†^
Targeted therapy or IO (yes vs no)	0.300	0.709	0.393–1.277	0.351
Comorbidities (yes vs no)	0.213	1.116	0.736–1.692	0.606
Stage (metastatic vs nonmetastatic)	0.304	1.458	0.804–2.645	0.214

† Statistically significant results.

IO: Immunotherapy; RR: Relative risk; SE: Standard error.

## Discussion

This study showed 85.2% seropositivity in cancer patients, whereas this rate was 97.5% in non-cancer controls. Additionally, IgG antibody titers in cancer patients were significantly lower than in the controls. The factors significantly associated with low seropositivity rates in the patient group were age and active chemotherapy. When the side effects in both groups were compared, the control group reported significantly more side effects after the first dose. Nevertheless, there was no significant difference between the groups in side effects after the second dose. Our findings confirmed the efficacy and safety of CoronaVac in cancer patients.

The COVID-19 pandemic negatively affected cancer patients. In addition to the severe course of COVID-19 in cancer patients, covidophobia, delays in cancer diagnosis and disruptions to oncological treatments increased the mortality of cancer patients during the pandemic [4–11]. NCCN and other oncological societies recommended that all cancer patients, especially those receiving active treatment, should be vaccinated as a priority [15]. The high seropositivity rate of cancer patients in our study also supports these recommendations, even though the seropositivity rate was relatively lower than in non-cancer adults.

The low seropositivity rate in cancer patients compared with the non-cancer controls found in this study was expected, as immunosuppression negatively affects the immune response. Similar to our results, Ariamanesh *et al.* found that older age, chemotherapy and hematological malignancies were related to lower seropositivity rates after administration of inactivated vaccine [20]. Massarweh *et al.* reported that chemotherapy plus immunotherapy treatment was associated with lower IgG titers in cancer patients vaccinated with the BNT162b2 mRNA vaccine [21]. Furthermore, studies evaluating the response to pneumococcal and influenza vaccines in patients with malignancy showed a decreased response in patients with hematological malignancies [24]. In another study, influenza vaccine response was low in breast cancer patients receiving active chemotherapy [25]. Our findings also highlight the negative effect of active treatment on immune response.

Although a clear relationship has not yet been established between antibody levels and prevention of the disease, the main target of the vaccines is to trigger the formation of neutralizing antibodies against the SARS-CoV-2 spike protein [26]. Harvey *et al.* reported an approximately tenfold increase in positive nucleic acid amplification test results among patients with positive antibody tests compared with those who had negative antibody tests, suggesting a protective effect of antibodies [27]. Another study demonstrated that the antibody titers were correlated with protection against COVID-19 [28]. Considering that the cellular immune response is suppressed in cancer patients, even adequate antibody levels may not effectively protect from the infection. Based on this, the application of additional doses, especially in cancer patients, may come to the fore in light of future studies. Patients receiving active chemotherapy and those in older age groups might be among the priority groups.

Another finding of our study was that the control group reported side effects more frequently, especially after the first dose. The reason might be that cancer patients experience such side effects due to the disease itself and their treatment processes, even before vaccination. The frequency of side effects reported after the second dose was found to be similar in both groups; this can be explained by the decrease in the perception of the side effects following the second dose.

Finally, when we created two groups by matching the patient and control groups by age and gender, the significant difference in seropositivity rates between the groups persisted.

This study had some limitations. First, we measured only spike IgG antibody levels of the participants but did not assess neutralizing antibody levels. However, studies have shown that neutralizing antibody levels are correlated with spike IgG antibody levels [29]. Second, we did not evaluate the pre-vaccination antibody levels of the participants. Nevertheless, we excluded patients who had a documented COVID-19 infection at any time before enrollment.

The median follow-up period after vaccination was 3 months, and eight patients were infected with COVID-19 during this period. The patient group will be followed up for long-term results to evaluate the effect of vaccination and antibody levels on disease prevention.

## Conclusion

This study highlighted the efficacy and safety of CoronaVac in cancer patients. The seropositivity rate was lower in cancer patients than in non-cancer controls, especially in patients aged over 60 years and those receiving active chemotherapy. Further studies with larger sample sizes are needed to determine the effective vaccine type and vaccine dose for cancer patients so that cancer patients might be protected from COVID-19-related morbidity and mortality without disrupting their oncological treatments.

**Summary points**

- COVID-19 is associated with high morbidity and mortality in cancer patients, but there are limited data on the efficacy and safety of currently used COVID-19 vaccines in cancer patients.
- We compared the seropositivity rate of cancer patients with non-cancer controls after CoronaVac administration and evaluated the factors affecting seropositivity in cancer patients.
- 776 cancer patients and 715 non-cancer volunteers who received a second dose of inactivated vaccine in 4–6 weeks were included in the study.
- The seropositivity rate and antibody levels were significantly lower in the patient group than in the non-cancer controls (p < 0.001). Age and chemotherapy were associated with lower seropositivity in cancer patients (p < 0.001).
- Side effects reported after the first dose were significantly higher in the control group (p = 0.001). There was no significant difference between the two groups after the second dose.
- The high seropositivity rate of cancer patients indicates that these patients benefit from the vaccine as protection from COVID-19 infection.
- It should be kept in mind that patients over the age of 60 and receiving chemotherapy have lower seropositivity rates and are in a higher risk group for COVID-19.
